# Supplementary material for: SpatioMark: quantifying the impact of spatial proximity on cell phenotype
Source: Bioinformatics. 2025 Jul 17;41(8):btaf409. doi: 10.1093/bioinformatics/btaf409 (PMC12343047; doi:10.1093/bioinformatics/btaf409)
Supplement: btaf409_Supplementary_Data [file btaf409_supplementary_data.pdf]

Supplementary Material

**Initial cell type**	**New cell type**
CD163+ Macrophages	Macrophages
CD68+ GranzymeB+ Macrophages	Macrophages
CD11b+ CD68+ Macrophages	Macrophages
CD68+ Macrophages	Macrophages
CD68+ CD163+ Macrophages	Macrophages
CD45RO+ CD4 T cells	CD4 T cells
GATA3+ CD4 T cells	CD4 T cells
CD4 T cells	CD4 T cells

Supplementary Table 1: Transitionary cell types in the original Schurch et al. manuscript which have been collapsed into a single cell type.

markers	status
CD68	cell type
CD8	cell type
CD45	cell type
CD4	cell type
CD20	cell type
CD11b	cell type
CD56	cell type
CD11c	cell type
CD3	cell type
CD44	cell state
FOXP3	cell state
p53	cell state
GATA3	cell state
T-BET	cell state
β-Catenin	cell state
HLA-DR	cell state
PDL1	cell state
Ki67	cell state
CD45RA	cell state
CD21	cell state
MUC1	cell state
CD30	cell state
CD2	cell state
Vimentin	cell state

LAG3	cell state
Na^+/K^+ ATPase	cell state
CD5	cell state
IDO1	cell state
Cytokeratin	cell state
aSMA	cell state
BCL2	cell state
CD25	cell state
PD1	cell state
GranzymeB	cell state
EGFR	cell state
VISTA	cell state
CD15	cell state
iCOS	cell state
Synaptophysin	cell state
GFAP	cell state
CD7	cell state
ChromograninA	cell state
CD163	cell state
CD45ro	cell state
CD31	cell state
Podoplanin	cell state
CD34	cell state
CD38	cell state
CD138	cell state

CDX2	cell state
Collagen IV	cell state
CD194	cell state
MMP9	cell state
CD71	cell state
CD57	cell state
MMP12	cell state

Supplementary Table 2: Classification of markers as either “cell type” or “cell state” used for partial ROC curve analysis in Figure 3. These designations were based on general biological knowledge and typical usage in the literature, including prior studies such as Schürch et al. (2020). We acknowledge that these designations are context-dependent and not consistent across studies. We provide these labels to make our assumptions transparent and to guide interpretation of spillover-related findings.

Interaction	Coefficient	Standard Error	p-value	Adjusted p-value
Na^+/K^+ ATP-ase expression in Tumours	0.240	0.06	5.7×10^{-5}	0.03
CD45RA expression in Tumours	0.038	0.07	0.59	0.78
Tumour proportions	2.6	1.2	0.033	0.099
Macrophage proportions	8.1	3.3	0.014	0.096
Smooth Muscle proportions	3.5	2.0	0.084	0.19
Na^+/K^+ ATP-ase expression in Tumours near Macrophages	-0.28	0.064	1.4×10^{-5}	0.017
CD45RA expression in Tumours near Smooth Muscle	0.89	0.22	6.0×10^{-5}	0.033

Supplementary Table 3: Survival model coefficients, standard errors, p-values, and adjusted p-values for different interaction types, from markers in cell types to cell type proportions and SpatioMark features.
